# 

**Published:** 1847-09

**Authors:** 


[Notices of the New Works are deferred until the next number.]

